# Single-Stage Bilateral Hip Reconstruction for Kabuki Hip Dysplasia in a Four-Year-Old: A Case Report

**DOI:** 10.7759/cureus.104161

**Published:** 2026-02-24

**Authors:** Abshina Shajahan, Mariam H Almuqeem, Assad Qureshi

**Affiliations:** 1 College of Medicine, Department of Orthopaedic Surgery, University of Sharjah, Sharjah, ARE; 2 Department of Family Medicine, Emirates Health Services, Sharjah, ARE; 3 Department of Orthopaedic Surgery, American Hospital Dubai, Dubai, ARE

**Keywords:** bilateral hip reconstruction, hip arthrogram, hip dysplasia, kabuki syndrome, pelvic osteotomy

## Abstract

Kabuki syndrome (KS) is a rare congenital syndrome characterized by distinctive facies, joint hyperlaxity, and hypotonia. When hip dysplasia manifests, it is usually severe, demonstrating treatment resistance with a recognized risk of later re-dislocation. Limited case numbers with variable outcomes compound uncertainty regarding optimal management. To date, all surgical cases described in the literature have been treated with open reduction, with no documented cases of concurrent bilateral hip surgery. Here, we present the case of a four-year-old boy with Kabuki syndrome who has been unable to walk since the age of three, following a spontaneous left hip dislocation. Clinical examination revealed characteristic facies, hypotonia, and painful and reduced left hip movements. Radiographs demonstrated left hip dislocation and severe bilateral acetabular dysplasia. Arthrographic evaluation demonstrated no barriers to left hip concentric reduction on abduction. Profound acetabular dysplasia was identified as the main structural abnormality causing dislocation. Single-stage bilateral hip reconstruction was undertaken without performing an open reduction of the left hip. Bilateral femoral derotation-shortening osteotomies and Dega pelvic osteotomies were performed in a single stage. A hip spica was applied for six weeks before commencing mobilization. One year postoperatively, the child was weight-bearing. Both hips remained concentrically reduced with improvement in dysplastic features. This report describes the successful management of hip dysplasia secondary to Kabuki syndrome using a novel approach. Open reduction with capsular plication of the dislocated hip was obviated, and both hips were corrected in a single operation with satisfactory clinical and radiological outcomes at one year.

## Introduction

Kabuki syndrome (KS), first described in Japan in 1981, is a rare genetic disorder characterized by abnormal facies, alongside other congenital anomalies. These include skeletal abnormalities, mild to moderate intellectual disability, and postnatal growth deficiency [1-3]. While Kabuki syndrome has an estimated prevalence of 1/32,000 in Japan, increasing reports from diverse ethnic groups globally suggest that the condition is underdiagnosed outside of Japan [3]. KS is typically sporadic, although familial cases have been reported with inheritance believed to be autosomal dominant or X-linked recessive [2,3].

Musculoskeletal abnormalities in KS include hip dysplasia, often manifesting as progressive hip joint dislocation, scoliosis, cervical ribs, and congenital hand anomalies [4,5]. The prevalence of hip dislocation has been reported to range from 18% to 62%, while the incidence of hip dysplasia is reported to be 12% in the neonatal period, suggesting that both developmental and congenital factors play a role. The limited number of studies, compounded by underdiagnosis of KS, has made it challenging to establish a standardized treatment protocol for hip dislocation in KS. Previous studies have demonstrated initially successful treatment of KS hip dislocation with bracing in a Pavlik harness or closed reduction under general anesthesia [1,2]. However, a significant number of these treated hips went on to sublux or re-dislocate, typically after walking age, supporting a progressive nature of the underlying condition. In patients with re-dislocations or those presenting after walking age, surgical treatment is often required. Open reduction of the hip has been undertaken in isolation or combined with femoral and pelvic osteotomies. Ligamentous laxity and severe acetabular dysplasia exhibited in this syndrome have been identified as contributory factors requiring correction [2].

All surgical cases reported in the literature have managed KS hip dislocation with capsular plication to address capsular laxity. Supplementary pelvic osteotomy has been proposed as the essential restraint to re-dislocation [1,2,6]. Previous studies have not addressed whether a dislocated hip could be corrected without open reduction and capsular plication, and whether single-stage surgery is feasible if both hips are dysplastic.

## Case presentation

A four-year-old boy with Kabuki syndrome presented to the pediatric orthopedic clinic at American Hospital Dubai for treatment of a left hip dislocation. He had been cruising but had not yet begun independent walking and stopped cruising shortly before three years of age, where X-rays from an external institute confirmed the hip dislocation. Hypotonia and characteristic facies were noted shortly after birth. The child also had cognitive impairment, with speech limited to sounds; these findings prompted us to refer him to a clinical geneticist, who performed genetic testing that confirmed the diagnosis of Kabuki syndrome. The parents reported that the child did not appear to be in pain from the hip dislocation; however, he refused to bear weight on the left leg.

**Figure 1:**
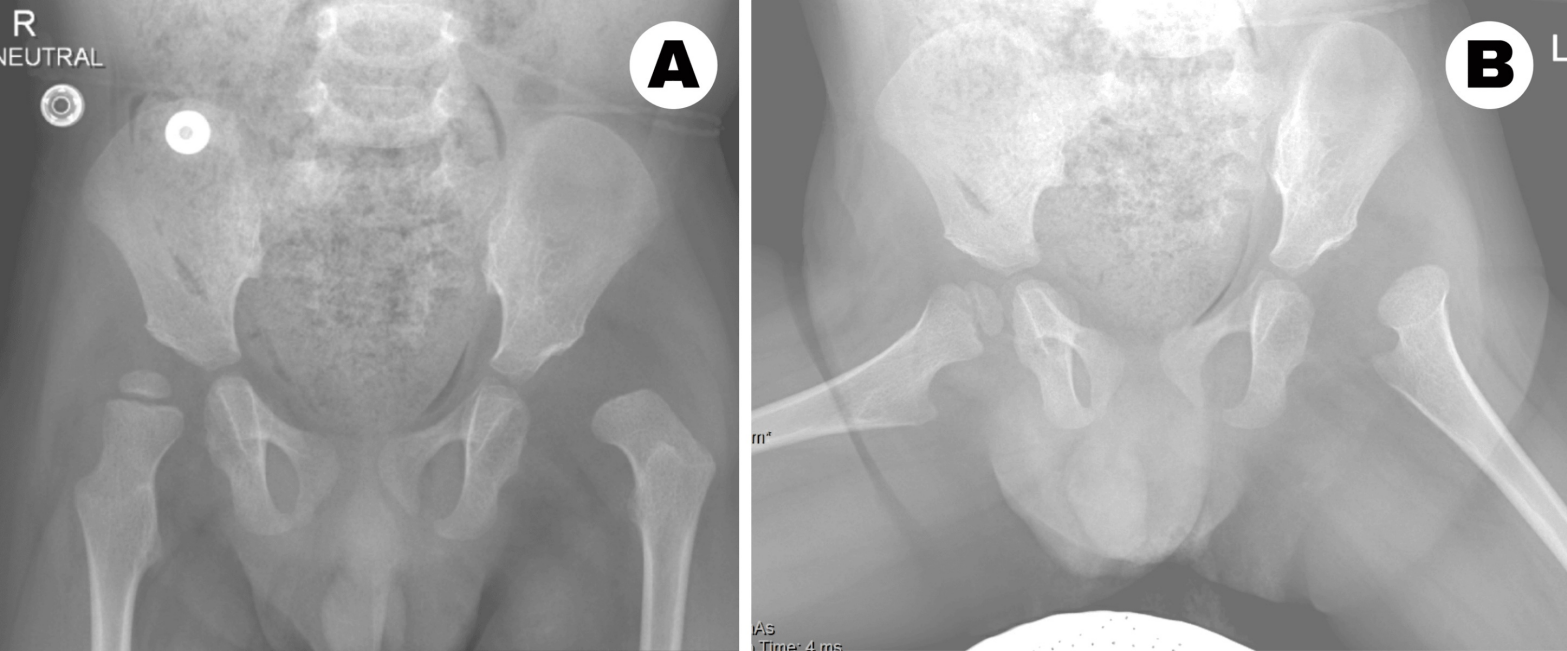
Preoperative anteroposterior pelvic radiographs (A) Anteroposterior pelvic radiograph demonstrating bilateral developmental dysplasia of the hip, with dislocation of the left hip and absence of the left femoral head ossific nucleus. (B) Anteroposterior pelvic radiograph obtained in maximal hip abduction showing persistent dislocation of the left hip.

On clinical examination, the patient exhibited dysmorphic facial features associated with Kabuki syndrome. He exhibited generalized hypotonia, particularly affecting the lower limbs, but demonstrated no contractures in the knees or ankles. The left leg appeared shorter, with restricted hip abduction. Flexion in the involved hip was limited to 90 degrees, with reduced abduction to 20 degrees compared with 50 degrees on the right. Radiographs revealed bilateral hip dysplasia, classified as moderate on the right and severe on the left. The left hip was dislocated, with an absent ossific nucleus at four years of age. The acetabular sourcil was short, with an acetabular index measuring 45 degrees. While the right hip was not frankly dislocated, it demonstrated 50% lateral uncoverage with a steep acetabular roof and an acetabular index of 35 degrees. Widening of the teardrop was evident in both hips (Figure 1).

Cortisol levels, thyroid function tests, echocardiography, and renal ultrasound were undertaken to exclude involvement of other organ systems, and all returned normal findings. A diagnostic arthrogram was undertaken under general anesthesia to define the anatomy and aid surgical planning. A spinal needle was inserted into each hip via a caudal approach under X-ray guidance, as described by Tönnis [7]. Contrast medium was injected into each hip to delineate the morphology of the femoral head and acetabular roof cartilage.

**Figure 2:**
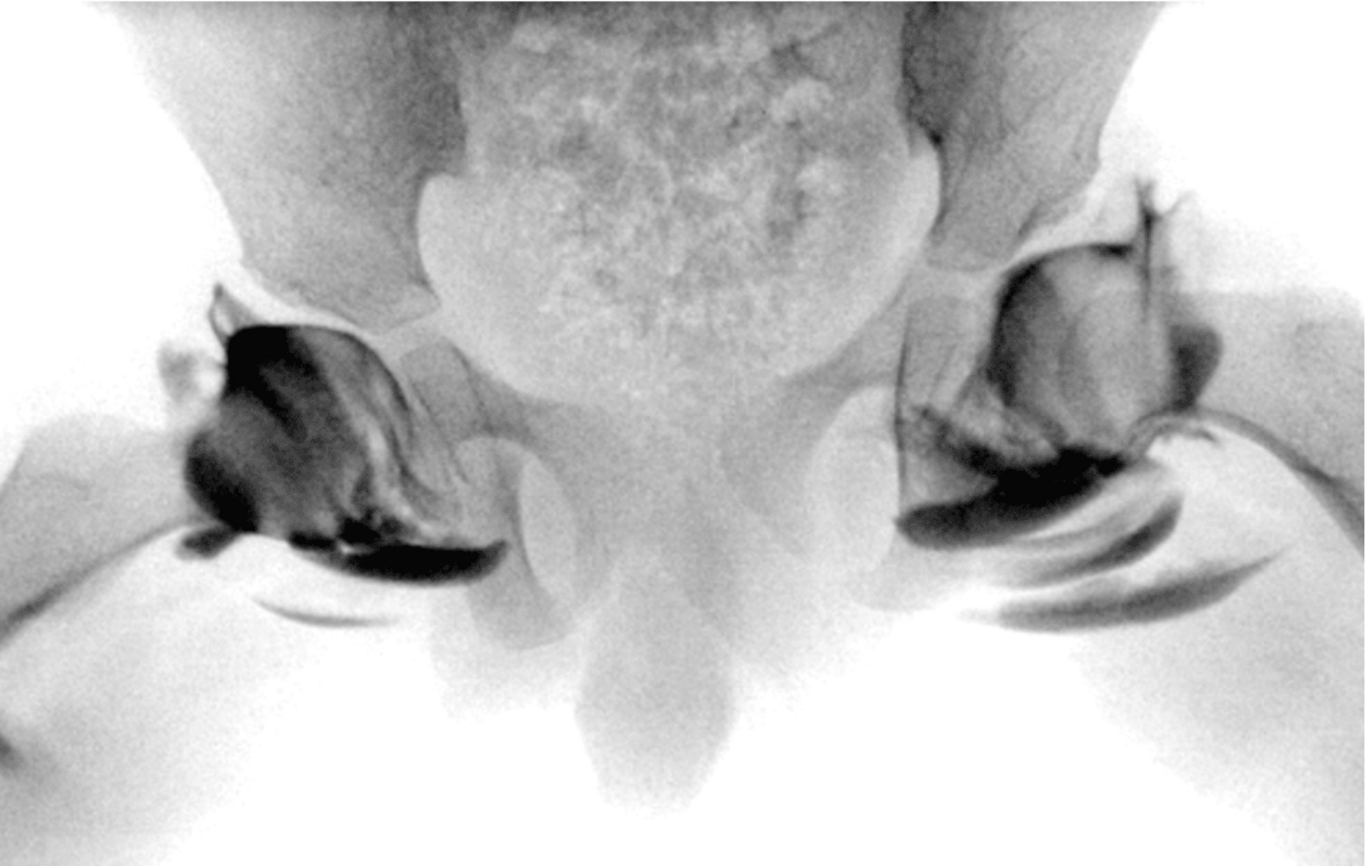
Anteroposterior arthrogram of the pelvis, with the left hip showing concentric reduction with abduction and internal rotation

The arthrogram of the left hip demonstrated that a concentric reduction could be achieved with abduction and internal rotation (Figure 2), confirming that there was no barrier to closed reduction in the left hip.

**Figure 3:**
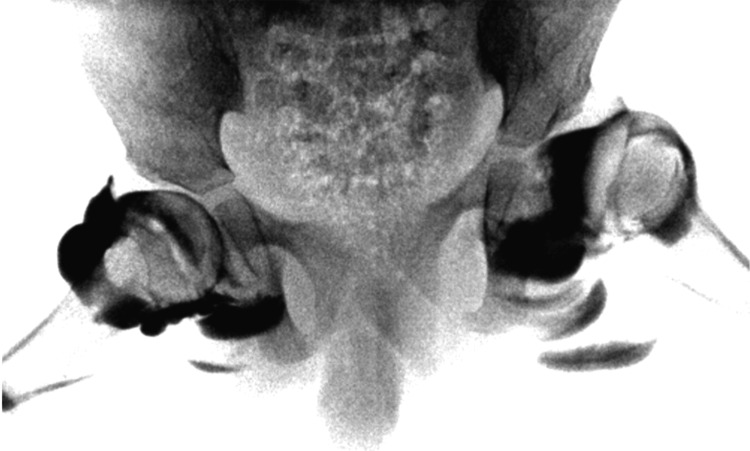
Anteroposterior arthrogram of the pelvis under external rotation stress: the left hip dislocates due to a thickened cartilaginous roof, while the right hip remains stable

External rotation caused superolateral displacement of the femoral head into a dislocated position (Figure 3). The arthrogram demonstrated that the left hip dislocation was facilitated by deformation of the thickened acetabular roof cartilage. The same maneuver in the right hip did not result in dislocation, as the cartilaginous roof was thinner on the right side (Figure 3).

Based on these findings, it was felt that an open reduction was not required to reduce the hip, as there were no anatomic barriers. Further, it was felt that capsular plication would not confer sufficient stability, given that acetabular deficiency was the primary cause of the dislocation rather than capsular laxity.

**Figure 4:**
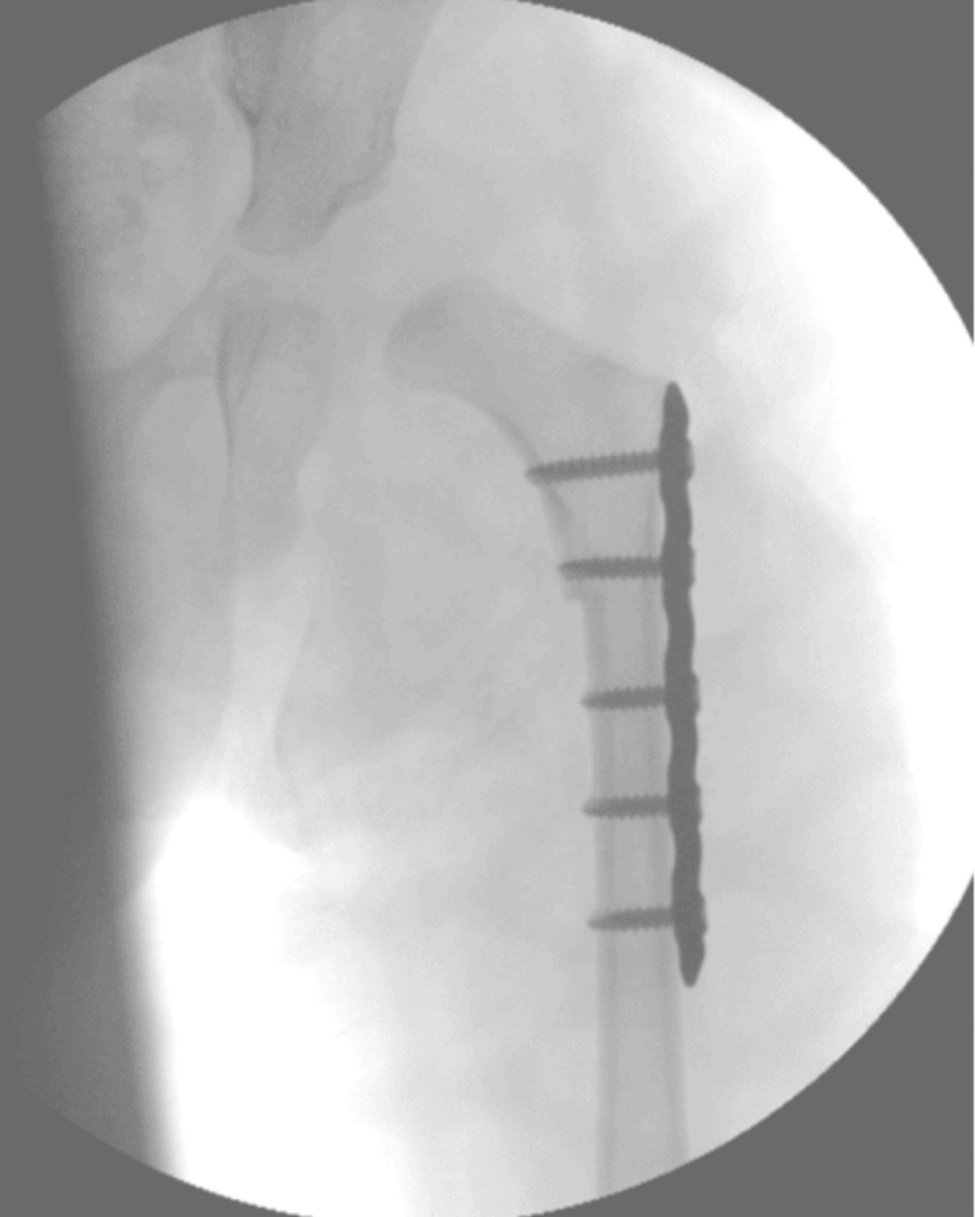
Anteroposterior intraoperative fluoroscopic image of the left hip and proximal femur following mild shortening and derotation osteotomy, fixed with a five-hole dynamic compression plate

The patient underwent single-stage, bilateral hip reconstruction by the senior author. The right hip was operated on first, followed by the left hip using the same technique. A lateral approach was used to expose the femur after elevation of the vastus lateralis. A five-hole, 3.5-mm dynamic compression plate was applied to the proximal femur. The proximal two plate holes were drilled, after which the plate was removed, and K-wires were inserted proximally and distally to guide rotation following the osteotomy. Transverse osteotomies were performed to excise a 2-cm block of femoral cortical bone. The shortening was intended to reduce pressure on the cartilaginous left femoral head once relocated. The secondary reason for shortening was to provide a superior cortical bone graft for the pelvic osteotomy compared with an iliac crest graft [8]. To achieve symmetry in leg length and rotational profile, the same shortening-derotation osteotomy was performed bilaterally. The distal segment was rotated from internal to external by 30 degrees to achieve optimal containment on intraoperative imaging with the femur in neutral rotation with the patella in a forward-pointing position. Varus was not introduced through the osteotomy, as it was felt that this may affect the abductor lever arm function during gait (Figure 4).

**Figure 5:**
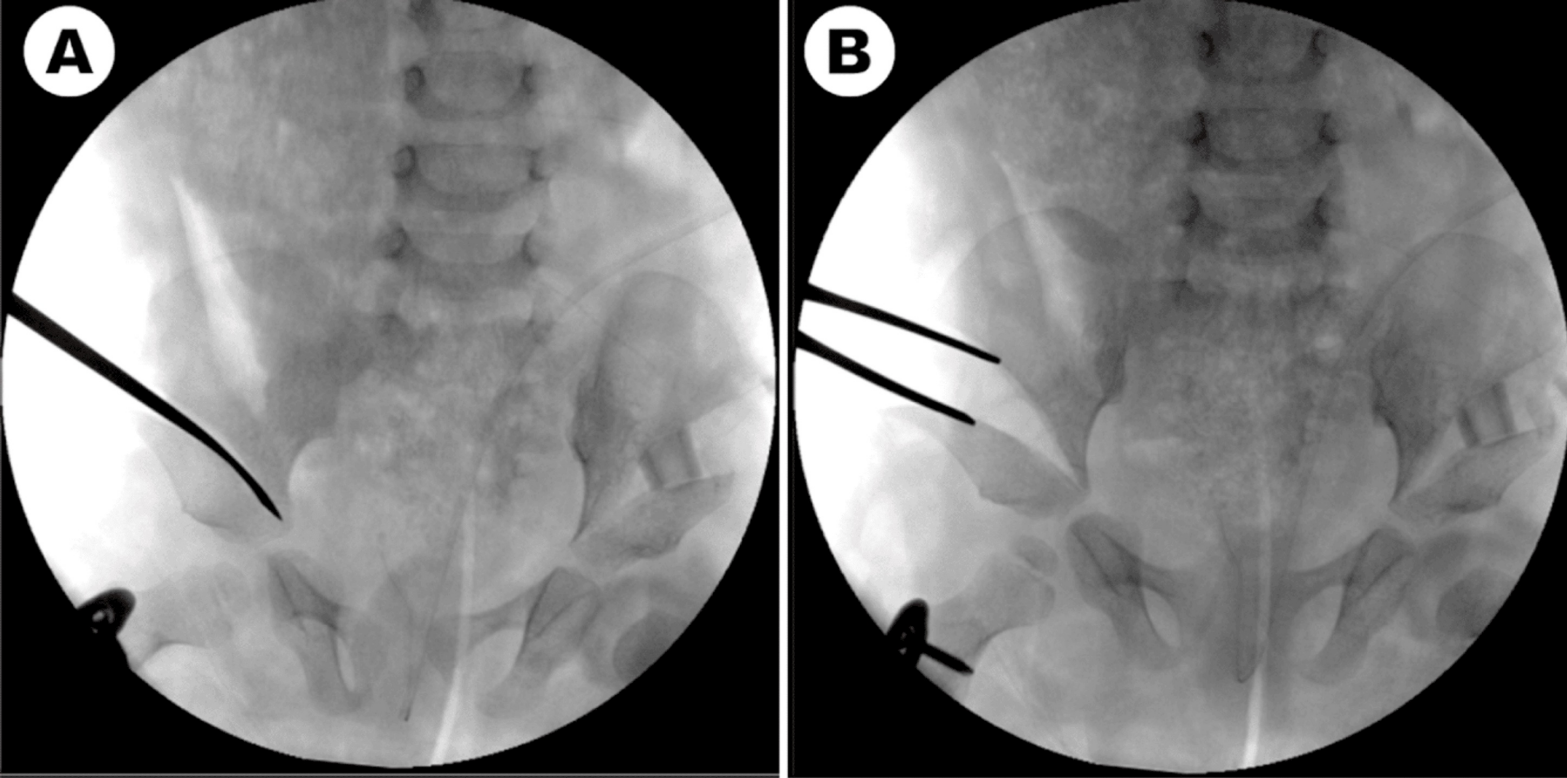
Anteroposterior intraoperative fluoroscopy image of the hip showing stages of pelvic osteotomy (A) Dega pelvic osteotomy performed on the right hip using an osteotome. (B) Right transiliac osteotomy distraction achieved using a laminar spreader.

A 7-cm “bikini” incision was made along the groin crease. The iliac crest apophysis was exposed and divided distal to the anterior inferior iliac spine. Straight and curved osteotomes were used to create an incomplete transiliac osteotomy (Figure 5A), which was distracted with a laminar spreader to produce the desired correction (Figure 5B).

**Figure 6:**
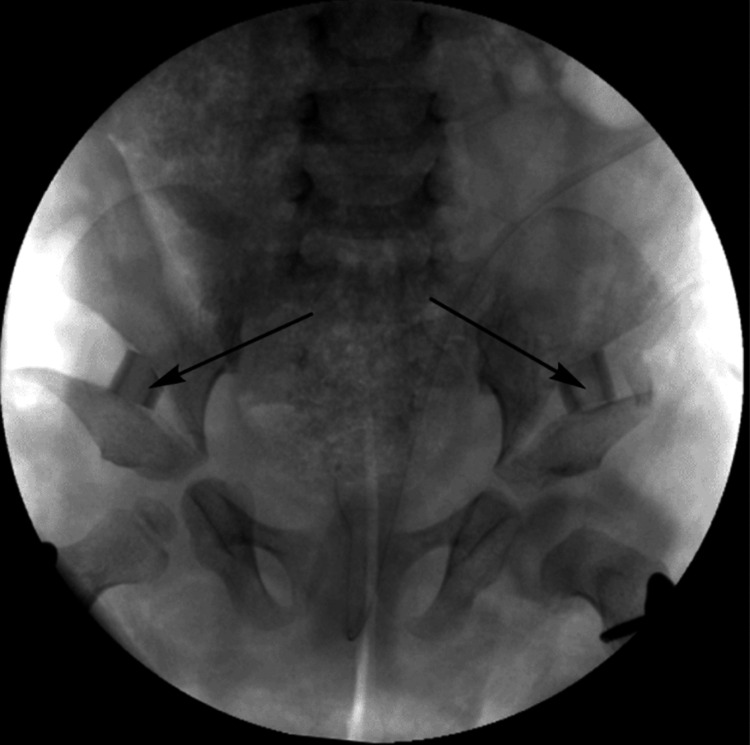
Anteroposterior intraoperative fluoroscopy of the hip showing the femoral segments (black arrows) placed in the transiliac osteotomy

The excised femoral segment was fashioned into a trapezoidal graft and placed deep into the osteotomy to maintain the correction (Figure 6).

**Figure 7:**
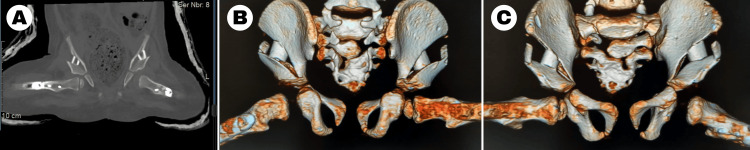
Postoperative day 2 CT images of the pelvis and proximal femora (A) Coronal CT image of the pelvis obtained with the hips in abduction. (B) Posterior 3D volume-rendered reconstruction of the pelvis obtained with the hips in abduction. (C) Anterior 3D volume-rendered reconstruction of the pelvis obtained with the hips in abduction.

The wounds were closed, and the child was placed into a padded hip spica cast for six weeks. A CT scan was performed on postoperative day 2 to confirm that both hips were concentrically reduced (Figure 7).

**Figure 8:**
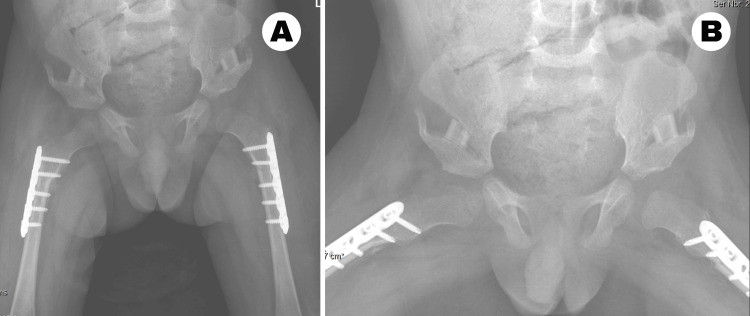
Postoperative follow-up X-ray at six weeks (A) Anteroposterior view of the pelvis and proximal femur at six weeks showing bilateral concentric hip reduction. (B) Anteroposterior view of the pelvis with hips in maximal abduction.

**Figure 9:**
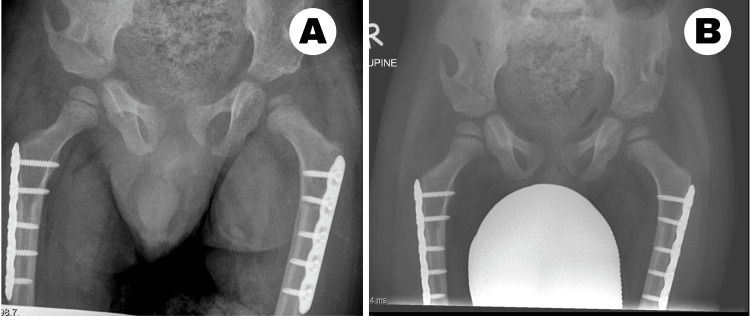
Postoperative follow-up X-rays of the pelvis and proximal femur at nine months and one year, respectively (A) Anteroposterior X-ray of the pelvis and proximal femur at nine months with both hips in joint, bilateral ossification of transiliac osteotomy sites, and left femoral epiphysis starting to ossify. (B) Anteroposterior X-ray of the pelvis and proximal femur at one year showing both hips in place, symmetrical femoral head ossification with thinning of the teardrop.

The cast was removed at six weeks, and the child was allowed to ambulate freely by crawling. The child was followed up at six weeks (Figure 8), nine months, and one year postoperatively (Figure 9) with radiographs.

Radiographs at each visit demonstrated that both hips were in the joint. The femoral epiphysis progressively ossified in the previously dislocated left hip. The acetabular index and center-edge angle were normalized. Subtle changes were evident in the lateral limb of the teardrop, indicating progressive deepening of the hip joint over the surveyed time points.

At one year postoperatively, the child was able to stand up from a sitting position and cruise while holding onto furniture. Due to hypotonia and the family being unable to obtain ankle-foot orthoses splints in the rural setting where they lived, he was unable to ambulate independently. Both femoral plates were removed under anesthesia at 13 months following surgery.

## Discussion

Kabuki syndrome is a rare disorder with orthopedic manifestations including hypotonia, ligamentous laxity, and hip dysplasia. When hip dysplasia is present, it is often severe and refractory to closed treatment. Hypotonia and ligamentous laxity are likely to contribute significantly to the pathogenesis of hip dysplasia [1,2]. Unlike idiopathic developmental hip dysplasia, which may demonstrate a favorable natural history with growth, hip dysplasia in Kabuki syndrome is often progressive [2]. Thus, failure to improve with standard age-appropriate treatment should raise suspicion of a syndromic cause of developmental hip dysplasia. Syndromic forms are also more likely to present with fixed dislocations and tend to require surgical intervention. Patients with non-idiopathic dysplasia of the hip, including those with Kabuki syndrome, often require more invasive surgical procedures, including open reduction and pelvic osteotomy, likely related to associated hypotonia and ligamentous laxity [9]. This was evidenced in the current case, where the left hip dislocated over time. Recurrent hip dislocations in older children exhibit a more unfavorable natural history, requiring more invasive treatment options. Wada et al. reported that open reduction alone may be insufficient to confer stability in the long term and that a pelvic osteotomy is crucial to reduce the risk of recurrent dislocation [2].

The current case report establishes several new facets to our understanding of the treatment of hip dysplasia in Kabuki syndrome. The use of arthrography to characterize the KS hip dysplasia and determine a surgical treatment plan has never been described in the literature. Two principal arthrographic findings defined the surgical treatment plan. First, a closed concentric reduction of the dislocated left hip was successfully achieved, eliminating the need for open reduction, which was therefore not performed. The second finding pertains to the arthrographic assessment of the acetabular roof and hip stability. In the dislocated hip, the severe acetabular dysplasia facilitated femoral head displacement, which was clearly visualized on the arthrogram. In contrast, the right hip could not be dislocated, as the thinner acetabular roof cartilage resisted deformation due to greater support from the underlying bony acetabular roof.

This concurs with the conclusions of Wada et al. that acetabular coverage was the chief factor governing stability of the dysplastic hip in KS [2]. In the current case, both capsular plication and its necessary preceding step, open reduction, were omitted from the surgical plan. To the author’s knowledge, this case represents the first time in the literature that surgery for a dislocated hip in KS did not include open reduction and capsular plication. This approach preserves the blood supply of the hip by avoiding capsulotomy. Additionally, omitting these steps reduced operative time and potential blood loss, enabling both hips to be treated in a single surgery. The right hip, although not dislocated at the time of surgery, was dysplastic with poor femoral head coverage, and its expected natural history would have been progressive dislocation. This case demonstrates that single-stage bilateral hip reconstruction in KS hip dysplasia is feasible and improves patient care, as only one period of spica cast immobilization was required rather than two separate surgeries necessitating double the period of immobilization.

**Figure 10:**
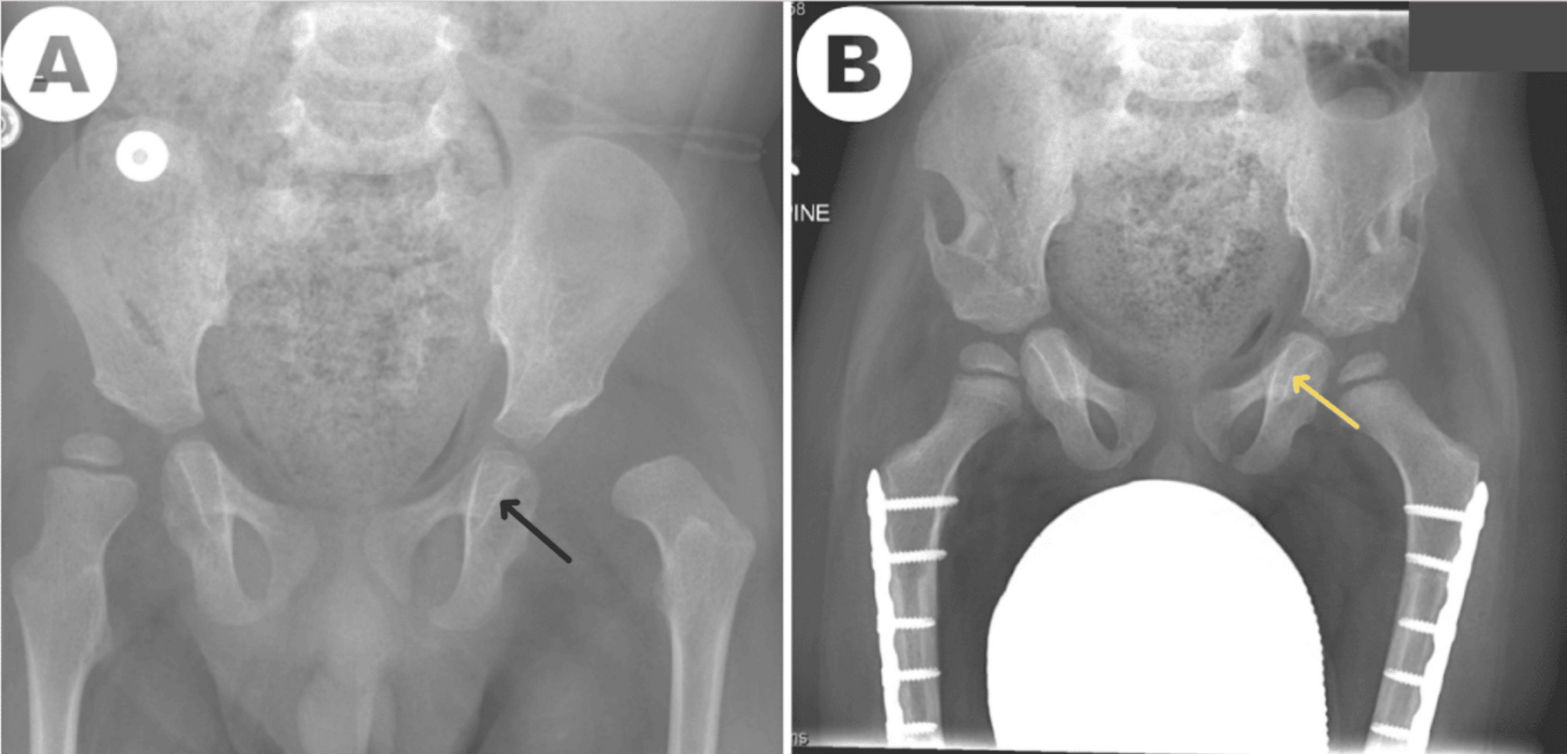
Anteroposterior X-ray of the pelvis and proximal femur showing preoperative versus one-year postoperative comparison of acetabular remodeling (A) Preoperative X-ray showing wide tear drop (black arrow), steep roof, and no ossification of the femoral head. (B) One-year postoperative X-ray showing narrow tear drop (yellow arrow), good femoral head coverage, and femoral head ossification.

Kabuki hip dysplasia can recur despite initially successful treatment, especially with increasing patient age at the time of surgery [1,2]. Late re-dislocation has been reported with persistent acetabular dysplasia despite pelvic osteotomy [2]. In the current case, we observed improvements in all radiographic parameters of hip dysplasia. The acetabular index and center-edge angle progressively normalized, indicating improved acetabular roof ossification and femoral head coverage. The left femoral head began to ossify despite remaining cartilaginous during the first four years of life. At one year, the femoral epiphyses demonstrated a symmetric appearance. Importantly, the postoperative radiographic appearance at one year, when compared with the preoperative radiographs, showed thinning of the teardrop in the dislocated left hip (Figure 10).

The absence of re-dislocation on the one-year follow-up X-ray has less prognostic significance than the progressive resolution of dysplastic features in the dislocated hip. We propose that teardrop responsiveness, indicating a deepening acetabulum, should be actively assessed on radiographs as a measure of surgical treatment success in Kabuki syndrome hip dysplasia.

## Conclusions

Kabuki syndrome is associated with severe hip dysplasia and progressive hip dislocation, which often recurs even after initial interventions. In line with existing literature, this case study emphasizes that incorporating pelvic osteotomy is essential for optimizing treatment outcomes. Our report provides novel insights into the management of this complex condition, specifically highlighting the utility of arthrography in identifying structural causes of dislocation and determining whether open reduction is necessary based on the presence of structural barriers.

Furthermore, we propose that an adequate pelvic osteotomy may eliminate the need for capsular plication. By adopting a surgical strategy that avoids capsulotomy and plication, clinicians can safely perform concurrent bilateral treatment for hip dysplasia in a single operative session. This approach potentially reduces the surgical burden on the patient while maintaining effective stabilization of the hip joints.
